# From general to specific: moving past the general population in the HIV response across sub‐Saharan Africa

**DOI:** 10.1002/jia2.25605

**Published:** 2020-10-01

**Authors:** Keletso Makofane, Elise M van der Elst, Jeffrey Walimbwa, Steave Nemande, Stefan D Baral

**Affiliations:** ^1^ FXB Center for Health and Human Rights Harvard University Boston MA USA; ^2^ Kenya Medical Research Institute Kilifi Kenya; ^3^ ISHTAR MSM Nairobi Kenya; ^4^ Evolve Yaoundé Cameroon; ^5^ Center for Public Health and Human Rights Department of Epidemiology Johns Hopkins School of Public Health Baltimore USA

**Keywords:** HIV, key and vulnerable populations, men who have sex with men, Africa, LGBT persons, general population

## Abstract

**Introduction:**

As the HIV field evolves to better serve populations which are diverse in risk and access to services, it is crucial to understand and adapt the conceptual tools used to make sense of the HIV pandemic. In this commentary, we discuss the concept of *general population*. Using a synthetic and historical review, we reflect on the genesis and usage of the *general population* in HIV research and programme literature, pointing to its moral connotations and its impact on epidemiologic reasoning.

**Discussion:**

From the early days of the HIV pandemic, the category of *general population* has carried implicit normative meanings. *General population* represented those people considered to be undeserving of HIV acquisition, and therefore deserving of a response. Framing the HIV epidemic in sub‐Saharan Africa as a generalized epidemic primarily affecting the *general population* has contributed to the exclusion of men who have sex with men from epidemic responses. The usage of this category has also masked heterogeneity among those it includes; the increasing focus on the use of interventions such as circumcision and HIV treatment as *general population* HIV prevention approaches has been marked by a lack of attention to heterogeneity among beneficiaries.

**Conclusions:**

We recommend that the term *general population* be retired from the field’s lexicon. HIV programmes should strengthen their capacity to describe the heterogeneity of those they serve and plan their interventions accordingly. To increase the efficiency and impact of the HIV response, it is urgent to stratify the category of general population by risk. Sexual networks are a promising basis for this stratification.

## 1 INTRODUCTION

The category of *general population* appears in policy briefs, research reports, guidance notes and policies. It punctuates the day‐to‐day speech of colleagues in global institutions and local programmes alike. It is as broad as it is ubiquitous, purporting to include a large swathe of society, if not the entire society, while helping funders, programmers, and researchers to make sense of the epidemic and choose among alternative courses of action. But despite its wide usage, the term is rarely explicitly defined. In the 2019 data report by UNAIDS [1], for instance, it garners several mentions without ever being linked to a glossary, neither in that document nor in the latest UNAIDS guideline on terminology [2]. Capacious as *general population* appears to be, in common usage it is clear what it is not: *general population* is an antonym for specific populations who require specific responses (or sometimes, as history proves, non‐responses). In this commentary, we reflect on the genesis and usage of the term in HIV literature, pointing to its moral connotations and its effects on epidemiologic thinking.

As the HIV field evolves to better serve populations which are diverse in risk and access to services, it is important to understand and adapt the conceptual tools used to make sense of the HIV pandemic. To help contextualize the articles in this special issue, we offer historical context for a concept that has fundamentally shaped the global HIV response yet is scarcely explicitly examined. Our hope is that grappling with this context will advance the development of conceptual tools better‐suited for understanding the contours of the current HIV pandemic.

We argue that framing HIV epidemics in sub‐Saharan Africa as *generalized epidemics* led to the wide‐spread understanding that they are almost exclusively heterosexual and homogeneous in nature. This is one of the mechanisms through which gay men and other men who have sex with men were excluded from the HIV responses mounted in the region by global, regional and national institutions. Crucially, this framing masked heterogeneity among those included in the *general population* category, weakening HIV responses for them. As an alternative to reporting and programming using the category of *general population*, we suggest using granular descriptions of distributions of risk.

## 2 DISCUSSION

### 2.1 The moral history of “General Population”

The idea of the *general population* made its appearance in early HIV research whose goal was to uncover the aetiology of a new and alarming syndrome. In a mid 1980s case series, for instance, Ioachim *et al*. [3] used the term to contextualize the incidence of lymphoma among gay men in New York, contrasting it with population‐wide, or *general population*, incidence. In the same period, Acheson *et al*. [4] used it to compare seroprevalence between two mutually exclusive groups: “high‐risk groups” and “general population,” distinguishing “special programmes” – interventions designed to “meet the needs of declared male homosexuals and persons who abuse drugs by injection” – from “material directed to the population as a whole,” [4] calling this latter group the *general population*. As these two examples illustrate, *general population* sometimes denoted a complement‐set and others a super‐set in relation to some group of interest. It sometimes was employed to make statistical comparisons and sometimes to distinguish and characterize groups of people. It was not defined explicitly and held unstable meaning, even within the same document. These ambiguities have proven persistent [5, 6, 7, 8, 9, 10] (See Appendix S1).

Among African people who had acquired the virus through heterosexual sex, the term was imbued with moral significance. Unlike the *risk‐groups* among whom the ravages of AIDS were first registered, *general population* represented those considered to be at *undeserved* risk [11]. According to Elizabeth Pisani, an architect of early UNAIDS guidance on HIV surveillance [12, 13, 14], it was an explicit goal to foster this moralistic understanding of risk for the purpose of igniting a global response: “… governments don’t like spending money on sex workers, gay men, or drug addicts… We had to find a way to translate the truth into something that governments might care about… Politicians are always happy to do nice things for innocent women and babies. Perhaps if we could show that doing nice things for injectors would protect innocent women and babies…” [15]. By the end of the 1990s, most of the bilateral investment in HIV programming was conducted under the assumption that HIV in Africa was transmitted nearly exclusively through heterosexual transmission and that “its primary impact [was] on the ‘general’ population” [11, 16]. Only in the 2000s did resources begin to be targeted at HIV programmes for men who have sex with men, though funding levels were grossly insufficient [17, 18, 19, 20].

In the context of the epidemic in the United States, social scientists had begun in the 1980s to examine the normative content of the distinction between *general* and *high‐risk*. Jan Grover, for instance, wrote in 1988 that according to the media, public health officials, and politicians “the general population is virtuously going about its business, which is not pleasure‐seeking (as drugs and gay life are uniformly imagined to be), so AIDS hits its members as an assault from diseased hedonists upon hard‐working innocents” [21]. The AIDS epidemic was understood to also be an “epidemic of signification,” [22] rapidly producing concepts that implicitly ascribe blame and innocence [23]. In these analyses, it was understood that categories used in scientific work are always entangled with already‐circulating cultural meanings [24]. In the context of wide‐spread homophobia, it followed that the categories used to understand the epidemic reflected widely held, negative attitudes towards gay men.

**Figure 1:**
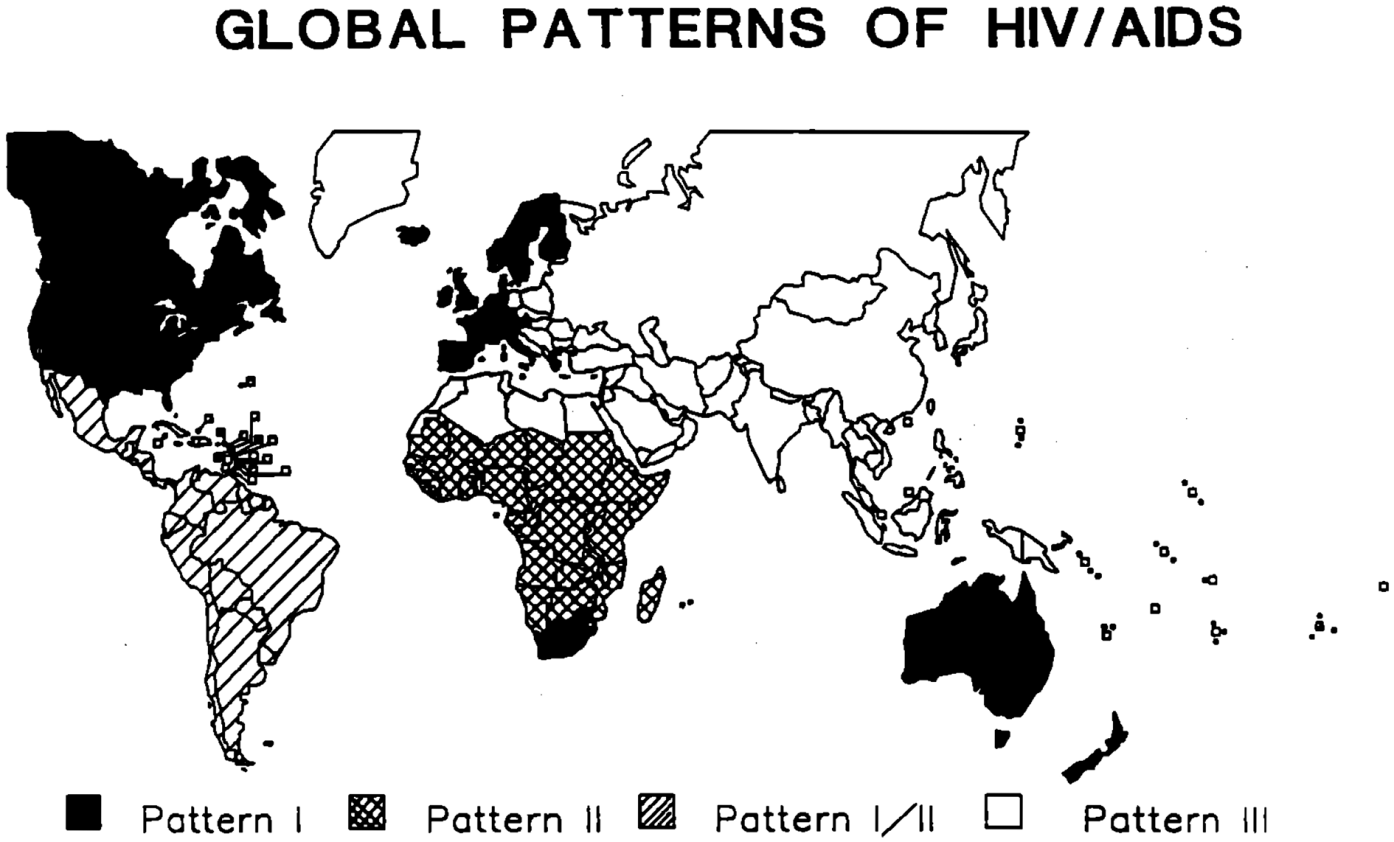
Global patterns of HIV and AIDS – 1989. The map shows Global patterns of HIV and AIDS according to the World Health Organization Global Programme on AIDS in 1989. *Pattern I* countries were those in which transmission was believed to occur predominantly among men who have sex with men and people who inject drugs; *Pattern II* countries were those in which “intravenous drug use and homosexual transmission [sic] are either non‐existent or occur at a very low level;” and *Pattern III* countries were those in which transmission was thought to have started later than in countries classified under *Pattern I* and *Pattern II*. This map appeared in a conference report published by the International Commission of Jurists [31].

The metaphors and meanings through which population distributions of disease are understood shape public health responses [25]. For the first two decades of the HIV response in sub‐Saharan Africa, men who have sex with men were ignored despite the emergence of the HIV epidemic among gay men in higher‐income settings and despite early evidence of its impact among gay men in other settings [11, 26, 27, 28]. There was early precedent. Citing mainly European studies of African immigrants and small‐sample studies in central Africa, an influential group of authors reported in 1986 that “African AIDS patients rarely report a history of homosexual activity or intravenous drug abuse” [29]. In the following years, this conjecture would reverberate through the World Health Organization’s communication about the global epidemic so that by the close of the 80s, it was the basis of a fundamental classification. Countries were grouped into three epidemic patterns, each pattern demanding a different kind of response (See Figure 1) [30, 31, 32]: *Pattern I* – those in which transmission happens predominantly among men who have sex with men and people who inject drugs; *Pattern II* – those in which “intravenous drug use and homosexual transmission [sic] are either non‐existent or occur at a very low level;” [31] and *Pattern III* – those in which transmission was thought to have started later than in countries classified under the first two patterns.

By the end of the 90s, the category of *Pattern II* had given way to *generalized epidemic* as the dominant way of understanding and describing HIV epidemics in sub‐Saharan Africa [12, 13]. The UNAIDS Guidelines for Second‐Generation HIV Surveillance, published in 2000, categorized HIV epidemics into three phases: low‐level, concentrated, and generalized. Generalized epidemics were initially defined using an HIV prevalence threshold of 1% in ante‐natal clinics [13]. Since then, the threshold was removed from surveillance guidelines, though the term *generalized epidemic* continues to be used.

Now, four decades and many lost lives since the start of the pandemic, the assumption that men who have sex with men do not feature in *generalized epidemics* has been roundly disproven [33]. All over sub‐Saharan Africa, LGBT‐led organizations provide services to men who have sex with men and advocate for programmatic inclusion in national, regional and global fora [34, 35]. While there has been considerable success in advocating for the establishment of targeted funding streams and inclusive normative guidance [36], governments and large agencies have struggled to shrug off their moralism. As men who have sex with men have been increasingly included in national HIV responses under the banners of Key Affected Populations, Most at Risk Populations, Key Populations and other risk groupings, it has sometimes been for explicitly instrumental ends. This is laid bare in AIDS national strategic plans in which it was the fear of contagion from *high‐risk* to *general* populations that gives impetus for interventions among the former [37]. This approach bears the imprint of global guidance, which cautions governments to pay attention to *high‐risk* groups in order to guard against spread “*into* the general population” [12, 14] rather than, for instance, dissemination *across* the population.

### 2.2 General population and epidemiologic reasoning

*General population* not only reflects the moral standing of those historically excluded from it, it shapes epidemiologic reasoning in relation to those it includes. Whereas in epidemiology the risk of an event – say acquiring HIV – is usually defined as the probability of the event’s occurrence, in the logic of the categorization into *general* and *high‐risk* populations, risk is implicitly defined through behaviour. In some instances, this creates contradictions. For example a person who has only had one sexual partner in her life – to whom she is married and with whom she has condomless sex – would be categorized as *low‐risk* or *general population*. On the other hand, if her partner were living with HIV and unsuppressed or if he were likely to acquire HIV from condomless sex with multiple other partners, then she would have an elevated risk (in the epidemiologic sense) of acquiring HIV. Under the scheme that divides people into *high‐risk* and *general*, the woman in this example would not be thought of as a member of some *high‐risk group*. By contrast, if a man who has only had one sexual partner in his life – another man – and had sex (condomless or not) with his partner, then they would both be considered members of a *high‐risk* group since their behaviour, sex with men, is considered risky in and of itself. The notion of risk that organizes this scheme focuses on behaviour, is shaped by sexual morals, and pays little attention to the most important factor in HIV transmission: the likelihood of sexual contact between someone who has acquired the virus and someone who has not.

This inattention has shaped HIV programming and research in sub‐Saharan Africa. As Baral *et al*. have previously argued [38], the focus of HIV treatment as *general population* prevention has not accounted for heterogeneity of risk among beneficiaries. The effectiveness of universal treatment at a population level is predicated on there being existing risk for HIV transmission between the recipient and their sexual contacts, yet little effort is made to understand the characteristics of these networks. Thus, transmission dynamics within networks might be the key to understanding why powerful, individual‐level HIV treatment effects have not translated into similarly powerful, population‐level incidence reductions. It might be that case that it is the size, composition and treatment coverage of personal sexual networks that determine population HIV prevention benefits.

The task of stratifying this category is urgent and of public health significance. According to UNAIDS, about 800,000 people in eastern and southern Africa acquired HIV in 2018 [1]. In one breakdown, 25% of new HIV infections were attributed to men who have sex with men, transgender people, sex workers, and the sexual partners of these groups and 75% were unattributed. In another, young women between the ages of 15 and 24 were said to account for 26%, and the remainder were unattributed. In both examples, the unattributed portion of new HIV infections, a portion containing much heterogeneity in risk, constitutes the majority.

Even within the categories of young women or men who have sex with men, however, there is considerable variation in risk. Among young women in Tanzania, for instance, having an older partner and engaging in transactional sex are each associated with double the HIV prevalence of not having an older partner and not having transactional sex respectively [39, 40]. In addition, there is substantial contact between groups [41, 42]. Past epidemiological studies among men who have sex with men in Botswana, Namibia, Malawi, South Africa, Kenya, Senegal and Nigeria suggest that sex with women is relatively common among men who have sex with men. The reported proportion who recently had sex with women has ranged from 20% to 75% [43, 44, 45, 46].

Epidemiologic and phylogenetic studies have consistently demonstrated the interconnectedness of the sexual networks of men who have sex with men with the networks of the remainder of the population [41, 47]. There remains, however, limited standard reporting of the attributable fraction of HIV epidemics across sub‐Saharan Africa secondary to the unmet needs of men who have sex with men. In part, the limited study and reporting of the population attributable fraction for HIV among men who have sex with men across sub‐Saharan Africa has emerged from the tacit assumption that they do not exist. Where they do exist, government consensus estimates often suggest such low population sizes so as not to be relevant for a comprehensive HIV response [48].

The urgency of obtaining a granular understanding of risk in the HIV epidemic is heighted by the fact that Africa is home to the largest ever generation of young people moving into adulthood [49, 50]. It is crucial to understand the diversity among youth, including sexual and gender identities and sexual practices, to design specific responses to address their actual unmet needs. Characterizing and celebrating that diversity increases the likelihood of differentiated programmes to adaptively scale to address the diverse needs of hundreds of millions of youth across sub‐Saharan Africa.

Geoffrey Rose is credited with the insight that, in a population, the highest burden of a disease in absolute numbers is to be found not among the few at highest risk, but the many with medium or low risk [51]. Following this line of reasoning, when mounting a public health response, it is the many that should be targeted to maximize impact, not the few. Dubbed the “population strategy” and manifested in the ideas of the *generalized epidemic* and the *general population*, this approach has animated HIV programming in sub‐Saharan Africa for the first two decades of the response.

But the population approach rests on assumptions that may not be met in reality: population interventions, if they depend on resources that are differentially distributed by risk, can widen disparities [52]. In addition, the largest number of cases might come from a small proportion of the population at extremely high risk [53, 54], rendering population‐wide interventions inefficient. Given the biology of HIV transmission intertwined with network‐level and social determinants of health, it has long been known that there are specific populations that bear a higher burden of risk and illness than others. Also because of social determinants, particularly stigma and discrimination operating at the interpersonal as well as at the structural level, the coverage of existing HIV prevention and treatment services and commodities follows the inverse care law: Those with greatest need have the lowest access to necessary services [55].

Learning from epidemics other than HIV would suggest the need to find an optimal balance between what Geoffery Rose termed *high‐risk strategies* – strategies that identify groups at higher risk and targets interventions appropriately – as opposed to a near exclusive focus on *population strategies*, as defined earlier. We must shift away from programming for the category of the general population to specific populations with specific needs. These may be defined by age, gender, labour migration, or geography at different scales. By tracing transmission clusters, phylodynamic modelling promises to aid our understanding of HIV transmission risk both within and across these populations [41, 42, 56]. In the absence of detailed sexual‐ or genetic‐network data, the determinants of sexual networks (e.g. micro‐geography, gender, occupation, mobility) should be used to stratify what are now termed *general* and *key* populations by risk. Interventions should be planned based on this stratification. The idea that the general population is a useful target for HIV surveillance and programmes should be replaced with a granular mapping of risk by the cross‐classification of multiple demographic variables. In conducting this mapping, the human rights of all individuals should be protected [57].

## 3 CONCLUSIONS

A shift away from the concept of *general population* suggests the need for understanding people’s individual needs, how these translate to a continuum of risks for HIV acquisition and transmission, and how these dynamically change over time in an epidemic. These considerations should be grounded in local context, even as valuable lessons are transmitted across countries and regions. For people who have thus far been classified as *general population* and those who have been classified as *key populations* alike, this means continually monitoring spatial and temporal patterns and identifying structural and behavioural causes of HIV transmission and HIV‐related ill health. It further means using this knowledge to produce tailored community‐led interventions including those that attend to structural determinants of health such as stigma and violence. Though it will take investment in research, there are programme strategies from which to draw inspiration. The approach of micro‐planning in sex worker programming, for example acknowledges that not all programme beneficiaries share the same risks or need the same programmatic responses [58]. Abandoning the category of *general population* affords a great opportunity to learn about the diversity of needs and adaptive strategies developed to respond to HIV among *key populations* [58, 59, 60, 61]. While likely necessary to advance the HIV response, this shift alone is not sufficient to overcome the structural determinants of the HIV epidemic. Perhaps, the only utility of the distinction between *general* and *key* populations is that it reinforces an understanding that intersecting stigmas, violence and criminalization play different roles for the HIV epidemic among these two categories. In moving away from the use of *general population* and towards studying why people are at risk for the acquisition and transmission of HIV in countries across sub‐Saharan Africa, the HIV response should follow with specific interventions, including structural interventions, to address those needs.

## COMPETING INTERESTS

None.

## AUTHORS’ CONTRIBUTIONS

KM led the development of the manuscript with specific sections led by SB. EvdE supported conceptualization of the manuscript and completed background research with JW. SN provided background materials for review. All authors provided overall direction of manuscript and inputs and text to specific sections of the manuscript.

## Supporting information


**Appendix S1.** Key Population/General Population Definitions.
