# Supplementary material for: From general to specific: moving past the general population in the HIV response across sub‐Saharan Africa
Source: J Int AIDS Soc. 2020 Oct 1;23(Suppl 6):e25605. doi: 10.1002/jia2.25605 (PMC7527769; doi:10.1002/jia2.25605)
Supplement: Supplementary file 1 — Appendix S1. Key Population/General Population Definitions. [file JIA2-23-e25605-s001.docx]

# Appendix S1. Key Population / General Population Definitions

## Key Populations are often considered to be distinct from the general population. Below is a selection of examples in abstracts from journal articles published from 2015 to 2019.

**Female sex workers in Kigali, Rwanda: a key population at risk of HIV, sexually transmitted infections, and unplanned pregnancy. Ingabire R et al. 2019.**

*Excerpt from abstract*: “Despite Rwanda's successes with preventing 70% of new infections in the general population through nationwide couples' testing in antenatal clinics, prevention and timely treatment in key populations including FSWs are lacking.” [7]

*Note*: (Heterosexual) couples’ testing services offered at ante-natal clinics are unlikely to reach men who have sex with men. That these are said to have reduced general population new infections suggests that men who have sex with men are not considered part of the general population.

**Informing HIV Prevention Programmes for Adolescent Girls and Young Women: A**

**Modified Approach to Programmatic Mapping and Key Population Size Estimation. Cheuk E. et al. 2019**

*Excerpt from abstract*: “With an increasing focus on adolescent girls and young women (AGYW) as a priority population for HIV prevention, programmes need to know the location of and how to effectively reach individuals who are at increased risk for HIV but were conventionally considered part of the general population.” [8]

*Note*: That there are individuals that were conventionally considered part of the general population implies that there are individuals that were not considered part of the general population.

**Getting to grips with the HIV epidemic in Russia. Stuikyte R et al. 2019.**

*Excerpt from abstract*: “The epidemic remains largely concentrated among key populations and their sexual partners but has the potential of affecting the general population.” [9]

*Note*: If key populations were considered part of the general population, then the epidemic would be understood to have already affected the general population.

**Gaps and opportunities: measuring the key population cascade through surveys and services to guide the HIV response. Hakim AJ et al. 2018.**

*Excerpt from abstract*: “With the inclusion of viral load testing, these surveys are able to monitor the entire treatment cascade among KP regardless of whether these populations access HIV services targeting the general population or KP.” [6]

*Note:* This implies that services for key populations are distinct from services for the general population, which implies that key populations are distinct from the general population.

## **Key populations and human rights in the context of HIV services rendition in Ghana. Laar A, DeBruin D. 2017**

*Excerpt from abstract*: “Given that they are bridging populations, whatever affects the Ghanaian key populations directly, affects the general population indirectly.” [5]

*Note*: This implies that key populations pose a contagion risk to the general population – a distinct population.

**Molecular tracing of heterosexual HIV-1 transmission in Georgia. Dvali N. et al. 2015.**

*Excerpt from abstract*: “HIV epidemic in Georgia has not spread to general population and remains concentrated around key populations at risk. Our work confirms that female sexual partners can serve as a bridge between key affected populations and general community, such as heterosexually active adults.” [10]

*Note*: That there is a bridge population implies that the “general community” is distinct form key populations.
